# RETRACTED: Wen et al. PTEN Deficiency Induced by Extracellular Vesicle miRNAs from *Clonorchis sinensis* Potentiates Cholangiocarcinoma Development by Inhibiting Ferroptosis. *Int. J. Mol. Sci.* 2024, *25*, 10350

**DOI:** 10.3390/ijms27104400

**Published:** 2026-05-15

**Authors:** Lijia Wen, Meng Li, Jigang Yin

**Affiliations:** State Key Laboratory for Diagnosis and Treatment of Severe Zoonotic Infectious Diseases, Key Laboratory for Zoonosis Research of the Ministry of Education, Institute of Zoonosis, College of Veterinary Medicine, Jilin University, Changchun 130062, China; wenlj21@mails.jlu.edu.cn (L.W.); mli20@mails.jlu.edu.cn (M.L.)

The journal retracts the article titled, “*PTEN Deficiency Induced by Extracellular Vesicle miRNAs from Clonorchis sinensis Potentiates Cholangiocarcinoma Development by Inhibiting Ferroptosis*” [1], cited above.

Following publication, concerns were brought to the publisher’s attention regarding the accuracy of the data presented in this article [1], as well as its substantial similarity to a previously published paper [2], which has since been retracted and was authored by a similar group of authors.

In accordance with standard journal procedures, an investigation was conducted by the Editorial Office and the Editorial Board, which identified concerns regarding the accuracy of the authorship list in comparison with the previously published article [2], as well as a failure to adequately disclose the prior publication and its subsequent retraction during the editorial process, as required under COPE guidance.

As a result, the Editorial Board has lost confidence in the reliability of the findings and has decided to retract this publication [1], as per MDPI’s retraction policy (https://www.mdpi.com/ethics#Updating_Published_Papers, accessed on 20 April 2026).

This retraction was approved by the Editor-in-Chief of the *International Journal of Molecular Sciences*.

The authors did not provide a comment on this decision.
